# Special Issue “Human Traits and Genomics: An Integrative Perspective”

**DOI:** 10.3390/ijms27114914

**Published:** 2026-05-29

**Authors:** Salvatore Saccone, Lidia Larizza, Giovanni Malerba

**Affiliations:** 1 Department Biological, Geological and Environmental Sciences, University of Catania, 95124 Catania, Italy; 2 Experimental Research Laboratory of Medical Cytogenetics and Molecular Genetics, IRCCS Istituto Auxologico Italiano, Via Ariosto 13, 20145 Milan, Italy; l.larizza@auxologico.it; 3 GM Lab, Department of Surgery, Dentistry, Paediatrics and Gynaecology, University of Verona, 37134 Verona, Italy; giovanni.malerba@univr.it

## 1. Introduction

The study of human traits has progressively evolved from classical Mendelian genetics to more complex models that integrate polygenic inheritance, environmental influences, and genome organization [1]. Despite the remarkable advances enabled by genome-wide association studies, genome microarray, and short- and long-read sequencing technologies (SRS and LRS), a substantial proportion of phenotypic variability remains unexplained [2,3]. Recent developments such as the human pangenome reference further expand our ability to capture population-level genomic diversity [4].

This observation suggests that genotype–phenotype relationships are shaped by multiple interacting layers, including coding and non-coding variation, epigenetic regulation, chromatin architecture, and environmental factors [5].

In this context, the present Special Issue offers an opportunity to reflect on how different approaches can contribute to a more comprehensive understanding of human traits. This perspective is consistent with the aim of this collection, which seeks to integrate diverse analytical strategies—from classical genetic approaches to advanced genomic and computational tools—to better elucidate the molecular basis of both Mendelian and complex traits.

The multi-layered nature of human trait genomics is schematically illustrated in the graphical abstract.

## 2. Integrating Genetic and Genomic Perspectives

Traditionally, rare pathogenic variants have been associated with Mendelian disorders, while common variants identified through GWAS have been linked to complex traits [1]. However, this distinction is becoming increasingly nuanced, as different classes of variants may jointly contribute to phenotypic variability [6,7].

In addition, it is now widely recognized that many functionally relevant variants are located outside coding regions, suggesting that regulatory mechanisms play a central role in gene expression and trait determination [8]. Furthermore, trans-regulatory effects contribute to the complexity of gene expression regulation [9], as well as genome organization and chromatin interactions [10].

Altogether, these observations support a shift towards more integrative models of genomics.

## 3. Insights from the Special Issue

The contributions included in this collection reflect different, yet complementary, aspects of human trait genomics.

Some studies focus on the identification and interpretation of genetic variants, ranging from rare mutations associated with specific disorders to population-scale analyses of clinically relevant genomic findings [11,12]. Other contributions emphasize the role of epigenetic mechanisms, highlighting how environmental and social factors may influence gene expression and take part in determining complex traits [13]. In parallel, the importance of genome organization emerges from studies investigating chromatin architecture and the functional impact of non-coding variants [14]. Finally, evolutionary and developmental perspectives provide a broader framework, linking genomic variation to biological processes across different levels of biological organization [15].

Together, these studies illustrate the diversity and complementarity of approaches currently applied to the study of human traits.

## 4. Emerging Themes

Several themes can be identified across the contributions. Human traits appear to be governed by a complex interplay of genetic variants with different frequencies and effect sizes [6]. At the same time, the functional relevance of non-coding regions and genome architecture is becoming increasingly evident [5].

The integration of multiple layers of information—genomic, epigenomic, and structural—represents a promising direction for future research [16], particularly for better explaining variability in human traits, including complex ones. In this context, computational approaches and artificial intelligence are likely to play an important role in data integration and interpretation [17,18].

Finally, the growing availability of genomic data is progressively enabling translational applications, particularly in the context of precision medicine [19].

## 5. Challenges and Perspectives

Despite these advances, several challenges remain unresolved.

The functional interpretation of genomic variants, especially in non-coding regions, is still incomplete [8]. In addition, the integration of different types of genomic data into coherent models represents a major analytical challenge. Addressing this complexity often requires multi-omics approaches—including genomic, epigenomic, and genome-wide chromatin analyses—applied across large cohorts and supported by robust bioinformatic frameworks.

From a clinical perspective, the implementation of genomic technologies requires robust workflows and standardized interpretation frameworks [20]. At the same time, cytogenetic/genomic approaches remain essential as first-line diagnostic tools to capture structural variation and genome architecture [21,22,23].

A broader evolutionary perspective may also contribute to a deeper understanding of genome function and its relationship with phenotypic diversity [24]. In this regard, studies conducted at a multi-ethnic scale will be particularly important to capture the full spectrum of human genomic diversity and improve the generalizability of findings.

Another important area concerns complex traits and neurodevelopmental disorders, where multiple genetic and environmental factors interact. In conditions such as autism spectrum disorders, integrative approaches are increasingly necessary to capture this complexity [22,25,26,27]. In this context, moving beyond gene-centric approaches towards network- and systems-level models will be essential to improve both diagnostic accuracy and biological interpretation.

Overall, further efforts are needed to integrate multi-omics data, improve functional annotation, and translate genomic findings into clinical practice.

## 6. Conclusions

The study of human traits is progressively moving towards more integrative frameworks that consider multiple layers of biological information, which will be especially relevant for explaining inter-individual variability and the architecture of complex traits. The contributions included in this Special Issue highlight the value of combining different approaches and perspectives and may help stimulate further research in this rapidly evolving field.
